# Supplementary material for: A Descriptive Analysis of the Relationship between Social Media Use and Vaccine Hesitancy among a Sample of Unvaccinated Adults in Canada
Source: Vaccines (Basel). 2022 Dec 7;10(12):2096. doi: 10.3390/vaccines10122096 (PMC9785008; doi:10.3390/vaccines10122096)
Supplement: Supplementary file 1 [file vaccines-10-02096-s001.zip › vaccines-2039812-supplementary.pdf]

Supplementary Material File S1 – Survey Instrument

HARVARD T.H. CHAN | SCHOOL OF PUBLIC HEALTH
Powerful ideas for a healthier world

If you intend to use this questionnaire for your project, please cite the publication and inform the authors by sending an e-mail to preparedness@hsph.harvard.edu

English version

1. Have you taken the COVID-19 vaccine? (Screening question)

- Yes, I received two or more doses
- Yes, I received one dose and the vaccine I took does not require a second dose
- I have received one dose of a two-dose COVID-19 vaccine and I plan to get my second dose
- I have received one dose of a two-dose COVID-19 vaccine but I am not sure if I will get my second dose
- I have received one dose of a two-dose COVID-19 vaccine but I do not plan to get the second dose
- Not yet, but I have an appointment scheduled
- No, and I do not have an appointment scheduled

2. What is your age?

- 18-24
- 25-34
- 35-44
- 45-54
- Over 54

3. What is your sex?

- Male
- Female

4. Are you a worker in any of the following categories? [select all that apply]

- Healthcare or social worker
- Residential facility worker
- Public health worker
- Correctional facilities worker
- Vaccine manufacturing or distribution worker
- Pharmacy worker
- Teacher or school staff
- Food processing worker

- Grocery store worker
- Postal and shipping worker
- Transportation worker
- Police or firefighter
- Volunteer
- Other

5. Please select your region of residence:

- Alberta
- British Columbia
- Manitoba
- New Brunswick
- Newfoundland and Labrador
- Northwest Territories
- Nova Scotia
- Nunavut
- Ontario
- Prince Edward Island
- Quebec
- Saskatchewan
- Yukon
- Other

6. What race/ethnicity do you consider yourself?

- White, Non-Hispanic
- Black, Non-Hispanic
- Asian, Non-Hispanic
- Two or more races
- Hispanic
- Prefer not to say
- Other, please specify: ___________

7. What is the highest level of schooling you have completed?

- Less than high school
- High school or equivalent
- Some college
- Bachelor's degree
- Post-graduate degree (i.e. Master, PhD, MD, etc)

- Other

8. Select the employment status that best describes your current situation [select one option only]:

- I am working - paid employee
- I am working - self-employed
- I am not working - on unemployment
- I am not working - on paid leave or furloughed
- I am not working - searching for work
- I am retired
- I am not working - on disability or worker's comp
- I am not working - and not looking for a job
- Other

9. Have you been diagnosed with COVID-19 by a healthcare worker or lab test result?

- Yes, over a year ago
- Yes, in the past 12 months
- No

10. Did any of your close family members or friends experience any of the following? [Check all that apply]:

- Tested positive for COVID-19 and had no symptoms or mild symptoms
- Tested positive for COVID-19 and had severe symptoms
- Died of COVID-19
- Lost their job or had a salary reduction due to COVID-19
- Got vaccinated for COVID-19 and had a positive experience
- None of my close family or friends experienced any of the above situations

11. How concerned are you about any of the following situations?

	Very concerned	Somewhat concerned	Not concerned
Contracting COVID-19 at work? (For example: your work settings that is not your home)			
Contracting COVID-19 outside of work? (For example: at the grocery store, when you are using transportation, or in other aspects of your daily life)			
Infecting your family or friends with COVID-19?			

12. How much time do you, on average, spend using social media?

- Every day, for 3 hours or more
- Every day, for more than 1 hour but less than 3 hours
- Every day, for less than 1 hour
- Alternate days
- Not often
- Never

13. From which sources did you get the most information about the COVID-19 vaccine? Select up to 3 sources:

- Local television news (on TV or on the web)
- English or French language national or cable network news (on TV or on the web)
- Non-English or French language television station (on TV or on the web)
- National newspaper (on paper or on the web)
- My town or other local newspaper (on paper or on the web)
- Non-English or French language newspaper (on paper or on the web)
- English or French language radio
- Non-English or French language radio
- News portal site such as Yahoo! or MSN
- Website of a government agency
- Social media
- Word of mouth
- Through my employer
- Other

14. How much do you trust the information you got so far about the COVID-19 vaccine?

- Not at all
- Very little
- Somewhat
- A lot

15. Did you get information about the COVID-19 vaccine from social media ? [check all that apply]

- No, I did not
- I am not sure
- Yes, from Facebook
- Yes, from Twitter
- Yes, from Youtube

- Yes, from Instagram
- Yes, from TikTok
- Other

16. If you were offered a COVID-19 vaccine - at no cost to you - how likely are you to take it?

- Very likely
- Somewhat likely
- I am not sure
- Somewhat unlikely
- Very unlikely
- I would not take it at the moment but would consider it later on

French version

1. Avez-vous reçu le vaccin contre la COVID-19?

- Oui, j'ai reçu deux injections ou plus
- Oui, j'ai reçu une injection et le vaccin que j'ai reçu ne nécessite qu'une seule injection
- J'ai reçu une dose d'un vaccin COVID-19 à deux doses et je prévois de recevoir ma deuxième dose
- J'ai reçu une dose d'un vaccin COVID-19 à deux doses mais je ne sais pas si j'obtiendrai ma deuxième dose
- J'ai reçu la première dose d'un vaccin COVID-19 à deux doses et je ne prévois pas de recevoir la deuxième dose
- Pas encore, mais j'ai un rendez-vous prévu
- Non, et je n'ai pas de rendez-vous prévu

2. Quel âge avez-vous?

- 18-24
- 25-34
- 35-44
- 45-54
- Plus de 54

3. Quel est votre sexe?

- Homme
- Femme

4. Appartenez-vous à l'une des catégories professionnelles suivantes? [cochez toutes les cases applicables]

- Professionnel de santé
- Agent en établissement résidentiel
- Professionnel de santé publique
- Agent d'établissement correctionnel
- Employé dans la fabrication ou la distribution de vaccins
- Préparateur en pharmacie
- Enseignant ou personnel scolaire
- Opérateur de transformation alimentaire
- Employé en magasin alimentaire
- Agent des services postaux
- Opérateur de transport
- Policier ou pompier
- Volontaire
- Autre

5. Veuillez sélectionner votre région de résidence

- Alberta
- Colombie-Britannique
- Manitoba
- Nouveau-Brunswick
- Terre-Neuve-et-Labrador
- Territoires du Nord-Ouest
- Nouvelle-Écosse
- Nunavut
- Ontario
- Île-du-Prince-Édouard
- Québec
- Saskatchewan
- Yukon
- Autre

6. De quelle race/ethnicité vous considérez-vous?

- Blanche (caucasienne), non hispanique
- Noire (africaine), non hispanique
- Asiatique, non hispanique
- Plus de 2 origines
- Hispanique
- Je préfère ne pas le préciser
- Autre

7. Quel est le niveau de scolarité le plus élevé que vous avez atteint?

- Inférieur aux études secondaires
- Études secondaires ou équivalent
- Études collégiales
- Baccalauréat
- Diplôme d'études supérieures (c.-à-d. Master, PhD, MD, etc.)

8. Sélectionnez le statut professionnel qui décrit le mieux votre situation actuelle [ne sélectionnez qu'une seule option]:

- En activité — salarié(e)
- En activité — indépendant
- Sans activité — au chômage

- Sans activité — en congé payé ou congé sans solde
- Sans activité — en recherche d'emploi
- Retraité
- Sans activité — en situation d'invalidité ou indemnisé pour accident du travail
- Sans activité — je ne recherche pas d'emploi
- Autre

9. Avez-vous été testé(e) positif(ve) à la COVID-19 par un professionnel de santé ou lors d'un test réalisé en laboratoire?
- Oui, il y a plus d'un an
- Oui, au cours des 12 derniers mois
- Non

10. Est-ce qu'un membre de votre famille ou un(e) ami(e) proche s'est trouvé(e) dans l'une des situations suivantes? [cochez toutes les cases applicables]:
- Testé(e) positif(ve) à la COVID-19 sans aucun symptôme ou des symptômes légers
- Testé(e) positif(ve) à la COVID-19 avec des symptômes sévères
- Décédé(e) de la COVID-19
- Ayant perdu son emploi ou subi une réduction de salaire à cause de la COVID-19
- Vacciné(e) contre la COVID-19 et mes amis et les membres de ma famille qui ont reçu le vaccin ont eu une expérience positive
- Aucun membre de ma famille ou ami(e) proche ne s'est trouvé dans l'une des situations décrites ci-dessus

11. Êtes-vous préoccupé(e) par l'une des situations suivantes?

	Très préoccupé(e)	Quelque peu préoccupé(e)	Pas préoccupé(e)
Contracter la COVID-19 au travail? (Par exemple: votre cadre de travail qui n'est pas votre domicile)			
Contracter la COVID-19 en dehors du lieu de travail? (Par exemple: à l'épicerie, dans les transports en commun, ou dans d'autres environnements de votre vie quotidienne)			
Contaminer votre famille ou vos amis avec la COVID-19?			

12. Combien de temps passez-vous en moyenne sur les réseaux sociaux?
- Tous les jours pendant 3 heures ou plus
- Tous les jours pendant plus d'une heure, mais moins de 3 heures
- Un jour sur deux

- Pas souvent
- Jamais

13. Quelle(s) source(s) vous a/ont transmis le plus d'informations sur le vaccin contre la COVID-19? Sélectionnez 3 sources maximum:

- Le journal télévisé local (à la télévision ou sur internet)
- Les actualités nationales ou du réseau câblé en anglais ou en français (à la télévision ou sur internet)
- Une chaîne de télévision non anglophone ou francophone (à la télévision ou sur internet)
- Les journaux nationaux (sur papier ou sur internet)
- Les journaux de ma ville ou autre journal local (sur papier ou sur internet)
- Les journaux non anglophones ou francophones (sur papier ou sur internet)
- Une radio de langue française ou anglaise
- Une radio de langue française ou non anglaise
- Un portail d'actualités tel que Yahoo! ou MSN
- Un site internet d'une agence gouvernementale
- Les réseaux sociaux
- Le bouche-à-oreille
- Par l'intermédiaire de mon employeur
- Autre

14. Dans quelle mesure faites-vous confiance aux informations que vous avez obtenues jusqu'à présent sur le vaccin contre la COVID-19? (SingleSelection)

- Pas du tout
- Très peu
- Un peu
- Beaucoup

15. Avez-vous obtenu des informations sur le vaccin contre la COVID-19 par l'intermédiaire des réseaux sociaux? [cochez toutes les cases applicables]

- Non, je n'en ai pas obtenu
- Je ne suis pas sûr(e)
- Oui — sur Facebook
- Oui — sur YouTube
- Oui — sur Instagram
- Oui — sur TikTok
- Oui — sur Twitter
- Autre réseau social

16. Si l'on vous proposait un vaccin contre la COVID-19 — sans frais pour vous — quelle serait la probabilité que vous l'acceptiez?

- Très probable
- Assez probable
- Je ne suis pas sûr
- Assez peu probable
- Très improbable
- Je ne l'accepterais pas pour le moment, mais je l'envisagerais ultérieurement
